# Emotionally Neutral Stimuli Are Not Neutral in Schizophrenia: A Mini Review of Functional Neuroimaging Studies

**DOI:** 10.3389/fpsyt.2016.00115

**Published:** 2016-06-22

**Authors:** Stéphane Potvin, Andràs Tikàsz, Adrianna Mendrek

**Affiliations:** ^1^Centre de recherche de l’Institut Universitaire en Santé Mentale de Montréal, Montreal, QC, Canada; ^2^Department of Psychiatry, Faculty of Medicine, University of Montreal, Montreal, QC, Canada; ^3^Department of Psychology, Bishop’s University, Sherbrooke, QC, Canada

**Keywords:** schizophrenia, functional neuroimaging, emotion, neutral, salience

## Abstract

Reliable evidence shows that schizophrenia patients tend to experience negative emotions when presented with emotionally neutral stimuli. Similarly, several functional neuroimaging studies show that schizophrenia patients have increased activations in response to neutral material. However, results are heterogeneous. Here, we review the functional neuroimaging studies that have addressed this research question. Based on the 36 functional neuroimaging studies that we retrieved, it seems that the increased brain reactivity to neutral stimuli is fairly common in schizophrenia, but that the regions involved vary considerably, apart from the amygdala. Prefrontal and cingulate sub-regions and the hippocampus may also be involved. By contrasts, results in individuals at risk for psychosis are less consistent. In schizophrenia patients, results are less consistent in the case of studies using non-facial stimuli, explicit processing paradigms, and/or event-related designs. This means that human faces may convey subtle information (e.g., trustworthiness) other than basic emotional expressions. It also means that the aberrant brain reactivity to neutral stimuli is less likely to occur when experimental paradigms are too cognitively demanding as well as in studies lacking statistical power. The main hypothesis proposed to account for this increased brain reactivity to neutral stimuli is the aberrant salience hypothesis of psychosis. Other investigators propose that the aberrant brain reactivity to neutral stimuli in schizophrenia results from abnormal associative learning, untrustworthiness judgments, priming effects, and/or reduced habituation to neutral stimuli. In the future, the effects of antipsychotics on this aberrant brain reactivity will need to be determined, as well as the potential implication of sex/gender.

## Introduction

Blunting of affect is one of the core negative symptoms of schizophrenia (1). In an effort to better understand this cardinal symptom, experimental studies have been performed using various types of emotional stimuli, including emotional images taken from validated databases. Decades of experimental research in the field have failed to confirm that emotional experience is reduced in schizophrenia as it would be expected from clinical observations. Instead, a meta-analysis of 26 studies using lab emotion induction paradigms revealed that schizophrenia patients tend to experience negative emotions (*aversion*) when presented with neutral or even positive stimuli (2). Likewise, another meta-analysis by Llerena et al. (3) showed that *arousal* ratings were increased in schizophrenia patients, compared to controls, when presented with neutral stimuli (3). In parallel to these behavioral studies, several functional neuroimaging studies have been performed in order to characterize the pathophysiological processes involved in the emotional disturbances associated with schizophrenia. The results of the neuroimaging studies are not always consistent with the aforementioned behavioral data, but fit with clinical observations. Thus, several studies have shown that frontal and limbic activations are reduced in schizophrenia patients presented with emotional stimuli (4), seemingly suggesting that this blunted brain reactivity of schizophrenia patients explains their emotional flattening. In 2012, Anticevic et al. (5) performed a meta-analysis of 35 functional neuro-imaging studies, which focused on the amygdala reactivity to emotional stimuli in schizophrenia. The amygdala obviously plays a key role in fear processing, and it had been hypothesized that abnormal amygdala activity could fuel paranoid ideation in schizophrenia (6). The meta-analysis from Anticevic et al. (5) showed that schizophrenia is associated with small reductions in amygdala activity in functional neuroimaging studies using explicit or implicit emotional images (faces or scenes). Importantly, a sub-analysis of the meta-analysis showed that this reduced amygdala activity is only present in studies examining the “negative emotion minus neutral” contrast, but not in the studies examining the “negative emotion minus baseline rest” contrast. In this latter case, there were no significant differences in amygdala activations between schizophrenia patients and controls. As observed by the authors, this subtle though important observation raised the possibility that schizophrenia patients may actually display hyper-activations in response to neutral stimuli, which could explain the apparent fronto-limbic hypo-activations that were initially reported.

Over the years, growing interest has been invested in studying how schizophrenia patients respond to emotionally neutral stimuli, using functional neuroimaging. To date, some studies have shown that the amygdala reactivity to neutral stimuli was increased in schizophrenia (7–12). Hyper-activations have also been observed in frontal, cingulated, and other limbic regions (13–18). Such results suggest that the previously reported fronto-limbic hypo-activations in schizophrenia patients in response to negative emotional stimuli may actually be explained by hyper-activations in response to neutral stimuli, as most functional neuroimaging studies examine relative activations during exposure to emotional vs. neutral stimuli. However, a minority of studies have failed to show that the brain reactivity to neutral stimuli is increased in schizophrenia (regardless of the brain region investigated) (19, 20). Given the uncertainty of findings, we sought to review the functional neuro-imaging studies in the field in order to identify the factors that could explain why some studies produce significant results, while others do not. Considering that the increased brain reactivity of schizophrenia patients to emotionally neutral stimuli is sometimes cited as one of the pillars of the highly influential aberrant salience hypothesis of psychosis (21), we also sought to briefly discuss, in the current mini-review, the various models that have been proposed to account for this intriguing phenomenon.

## Methods

Briefly, a search was performed in Pubmed, Embase, and Google scholar, using the following key words: “schizophrenia” or “psychosis” and “fMRI” or “PET” or “neuroimaging,” and “salience” or “emotion” or “neutral.” The references of all the studies included in the review were also screened. For the purpose of this mini-review, we only included studies on neutral stimuli investigated within the confines of studies on emotional processing, meaning that studies investigating other types of salience (reward, novelty, etc.) were not included, as well as studies using cognitive paradigms (e.g., episodic memory, etc.) using only neutral stimuli. Studies assessing complex emotional and cognitive interactions (e.g., emotional response inhibition, emotional memory, etc.) were also not included, as well as EEG and structural imaging studies. As shown in Table 1, 36 studies were retrieved, and the following factors were extracted: the population under study (including their age and sex), the type of imaging (positron emission tomography, PET vs. functional magnetic resonance imaging, fMRI), the analysis model (event-related or block design), the type of stimuli (faces vs. other), and the emotional processing instruction (implicit or explicit). Paradigms asking participants to passively view the emotional stimuli or to identify the gender of the displayed faces were classified as implicit paradigms, whereas paradigms asking participants to recognize emotional expressions or to rate their emotional responses to the emotional material were classified as explicit paradigms. Finally, we also specified the contrast that was used for the analyses (example: neutral vs. baseline rest) as well as the kind of search that was undertaken (whole brain vs. analyses based on regions of interest).

**Table 1:** **Brain responses to emotionally neutral stimuli in psychotic patients**.

Reference	Participants	Mean age (years); % of males	Task	Implicit/explicit	Event/block	Whole brain/ROI	Contrast	Increased activations	Decreased activations
**Schizophrenia**									
Bjorkquist and Herbener (13)	14 SCZ, 14 controls	31.6; 71	Positive, negative, and neutral IAPS images	Explicit	Block	Whole brain	Neutral vs. affective	Middle cingulate	
Dowd and Barch (22)	40 SCZ, 32 controls	36.8; 65	Positive, negative, and neutral pictures, words and faces	Explicit	Event	ROI	(*Post hoc*) neutral vs. baseline	No differences	
Fernandez-Egea et al. (7)	11 SCZ patients, 10 controls	28.6; 100	Happy, sad, and neutral faces	Explicit	PET	ROI	Control vs. baseline	Amygdala	
Habel et al. (14)	17 SCZ patients, 17 controls	34.4	Angry, fear, happy, neutral, and sad faces	Explicit	Event	Whole brain	Neutral vs. baseline	Cuneus, dorso-lateral prefrontal cortex, inferior parietal, middle frontal, middle orbito-frontal, postcentral gyrus, precentral gyrus, precuneus, putamen, subgenual anterior cingulate, superior occipital, superior parietal	Cuneus, fusiform gyrus, superior temporal gyrus
Hall et al. (8)	24 SCZ patients, 24 controls	Un-specified	Fearful and neutral faces	Implicit	Block	Whole brain and ROI (amygdala)	Neutral vs. baseline	Amygdala, fusiform gyrus, lingual gyrus, posterior cingulate	
Holt et al. (9)	15 SCZ, 16 controls	47.7; 100	Fearful, happy, and neutral faces	Implicit	Block	ROI	Neutral vs. baseline	Amygdala, hippocampus	
Holt et al. (23)	14 SCZ, 18 controls	42.9; 78.6	Pleasant, unpleasant, neutral sentence pairs	Explicit	Event	Whole brain	(*Post hoc*) neutral vs. baseline	Posterior cingulate gyrus	
Jensen et al. (24)	13 SCZ, 13 controls	37.6; 76.9	Classic conditioning (loud noise)	Implicit	Event	Whole brain	Neutral comparator vs. baseline	Hippocampus, middle cingulate, prefrontal cortex, thalamus, ventral striatum	
Lakis and Mendrek (25)	37 SCZ, 37 controls	32.5; 51	Positive, negative, and neutral IAPS images	Implicit	Block	Whole brain	Neutral vs. baseline	Amygdala, angular gyrus, middle frontal gyrus, middle temporal, superior orbitofrontal	Anterior cingulate, cuneus, mid cingulate, precentral gyrus, putamen
Lee et al. (19)	15 SCZ, 14 controls	31.7; 53.3	Positive, negative, and neutral IAPS images	Explicit (ambiva-lence task)	Event	ROI	(*Post hoc*) neutral vs. baseline	No differences	
Lindner et al. (26)	36 SCZ (25 flat affect), 40 controls	30.6; 63.9	Disgust, fear, happy, and neutral faces	Priming	Block	ROI	Fearful vs. neutral	Amygdala (only in patients with flat affect)	
Mier et al. (10)	11 SCZ, 16 controls	32.5; 64	Angry, disgust, fear, happy and neutral faces	Explicit	Event	ROI	(*Post hoc*) neutral vs. baseline	No differences	
Mier et al. (11)	16 SCZ, 16 controls	34.3; 68.8	Angry, fearful, happy, and neutral faces	Implicit	Event	ROI	Neutral vs. baseline	Amygdala, superior temporal sulcus	
Modinos et al. (27)	15 SCZ (FEP), 20 controls	27.9; 73.3	Positive, negative, and neutral IAPS images	Explicit	Event	Whole brain and ROI (amygdala)	Neutral vs. baseline	Amygdala, anterior insula, inferior frontal gyrus	
Mothershill et al. (15)	25 SCZ, 21 controls	42.9; 80	Angry and neutral faces (dynamic)	Implicit	Block	Whole brain	Neutral vs. baseline	Lack of deactivation of medial prefrontal cortex	Cerebellum^a^
Pankow et al. (28)	35 SCZ, 36 controls	31.1; 62.9	Positive, negative, and neutral IAPS images	Implicit	Event	ROI	(*Post hoc*) neutral vs. baseline	No differences	
Potvin et al. (16)	22 TR-SCZ vs. 24 SCZ, vs. 39 controls	TR-SCZ: 33.4; 72.7; SCZ: 31.3; 54.2	Positive, negative, and neutral IAPS images	Implicit	Block	Whole brain	Neutral vs. baseline	Dorso-medial prefrontal cortex (TR vs. SCZ and HC)	
Rauch et al. (29)	12 SCZ, 12 controls	27.7; 75	Happy, sad and neutral faces (priming)	Explicit	Event	ROI	Neutral face vs. erased faces	No differences	Amygdala (if neutral prime)
Reske et al. (30)	18 SCZ (FEP), 18 controls	31.9; 55.6	Happy, sad, and neutral faces	Explicit	Event	Whole brain	Emotions vs. baseline (with *post hoc* for neutral faces)	Posterior cingulate^a^	Anterior cingulate^a^, fusiform gyrus^a^, orbitofrontal
Romaniuk et al. (31)	20 SCZ, 20 controls	36.4; 70	Classic conditioning (IAPS pictures)	Implicit	Event	ROI	Neutral stimuli vs. averse stimuli	No differences	Midbrain
Schwartz et al. (32)	8 SCZ, 8 controls	52.1; 87.5	Fearful and neutral faces	Implicit	Block	ROI	Initial encoding vs. repeated encoding	Lack of reduction of the fusiform gyrus activation with repeated presentation of faces^a^	
Shin et al. (12)	16 SCZ, 16 controls	32.0; 100	Fearful, happy, and neutral faces	Implicit	Block	Whole brain and ROI (amygdala)	Neutral vs. baseline	Amygdala and superior orbitofrontal gyrus	
Surguladze et al. (18)	15 SCZ, 11 controls	43.1; 100	Fearful and neutral faces	Implicit	Event	Whole brain	Neutral vs. baseline	Para-hippocampal gyrus, fusiform gyrus	
Suslow et al. (33)	30 SCZ, 35 controls	30.9; 56.7	Angry, happy, and neutral faces	Priming	Event	ROI	(*Post hoc*) neutral vs. baseline	Amygdala (initial phase, neutral prime)	
Taylor et al. (34)	21 SCZ, 21 controls	40.7; 66.7	Angry, fearful, happy, sad, and neutral faces	Implicit	Event	Whole brain	Neutral faces: preference > gender identification	Hippocampus, insula, middle frontal gyrus, middle temporal gyrus and para-hippocampus	Middle cingulate gyrus
Taylor et al. (35)	23 SCZ patients, 15 controls	39.2; 73.9	Positive, negative, and neutral IAPS images	Explicit	Blocks	ROI	Neutral vs. baseline	No differences	Amygdala, medial prefrontal cortex
Taylor et al. (36)	14 SCZ, 13 controls	36.4; 71.4	Negative and neutral IAPS images	Explicit	PET	ROI	Neutral (non-aversive) vs. baseline	No differences	Amygdala
Ursu et al. (37)	20 SCZ, 20 controls	28.8; 75	Positive, negative, and neutral IAPS images	Explicit	Event	ROI	Neutral vs. baseline	No differences	
Whalley et al. (38)	15 SCZ, 14 controls	38.4; 73.3	Positive and neutral IAPS pictures	Explicit	Blocks	ROI	Neutral vs. baseline	Amygdala and hippocampus (non-significant trend)	
**Individuals at risk for psychosis**									
Barbour et al. (39)	19 SCZ offsprings, 25 controls	8–19; 63.2	Angry, fearful, happy, sad and neutral faces	Explicit	Event	ROI	(*Post hoc*) neutral vs. baseline	No differences	
Diwakar et al. (40)	19 CHR, 24 controls	14.3; 63.2	Negative, positive, and neutral faces	Explicit	Event	ROI	Neutral vs. baseline	No differences	
Modinos et al. (27)	18 UHR, 20 controls	24.4; 55.6	Positive, negative, and neutral IAPS images	Explicit	Event	Whole brain and ROI (amygdala)	Neutral vs. baseline	Anterior insula, inferior frontal gyrus (UHR and FEP vs. HC)	
Modinos et al. (41)	17 PLEs, 17 controls	19.8; 41.2	Negative and neutral IAPS images	Explicit	Event	ROI	Neutral vs. baseline	No differences	
Seifert et al. (17)	12 CHR, 12 controls	24.5; 83.3	Angry, fearful, happy, sad, and neutral faces	Explicit	Event	Whole brain	(*Post hoc*) neutral vs. baseline	Inferior frontal gyrus, superior frontal gyrus, and thalamus	
Van Buuren et al. (20)	24 SCZ siblings, 25 controls	29.4; 66.7	Positive, negative, and neutral IAPS images	Explicit	Blocks	Whole brain	Neutral vs. baseline	No differences	
Van der Velde et al. (42)	15 UHR, 16 controls	23.1; 53	Negative and neutral, IAPS images	Explicit	Event	Whole brain	Neutral vs. baseline	No differences	Posterior cingulate, temporal pole

*CHR, individuals at clinical high-risk for psychosis; FEP, First episode psychosis; IAPS, International Affective Pictures System; PET, positron emission tomography; PLEs, individuals with psychotic-like experiences; ROI, region of interest; SCZ, Schizophrenia; TR, treatment-resistant; UHR, ultra-high risk*.

*^a^Neutral and emotional images*.

## Main Results

A few patterns emerge from the results described in Table 1.

Out of 36 studies, 22 studies found brain hyper-activations in response to emotionally neutral stimuli in psychotic individuals (schizophrenia patients or individuals at risk for psychosis), relative to controls. Out of 29 studies in schizophrenia, 20 studies found brain hyper-activations, suggesting that the phenomenon is relatively robust in this population [Note: the study from Whalley et al. (38) found non-significant trends for the between-group difference; therefore, it will not be considered in the subsequent discussion of results]. By contrast, in individuals at risk for psychosis, only two studies out of seven found brain hyper-activations. Brain regions varied significantly across studies. The regions the most frequently found to be impaired were the amygdala (*n* = 10), prefrontal (*n* = 14) and cingulate sub-regions (*n* = 6), and the (para-) hippocampus (*n* = 6). The amygdala, hippocampus, and (anterior) cingulate gyrus are regions with well-established roles in emotion salience attribution (43, 44), and all have been consistently shown to be involved in the emotional disturbances associated with schizophrenia (4, 45).

Hyper-activations were found in studies using implicit as well as explicit emotion processing paradigm. The very fact that the aberrant brain reactivity of psychotic individuals to neutral stimuli was present in studies using implicit emotion paradigms suggests that this phenomenon does not result exclusively from impaired top-down cognitively controlled mechanisms. Notably, the failure to detect increased brain reactivity to neutral stimuli in schizophrenia was observed mostly in studies using explicit emotion processing paradigms (seven times out of nine) (19, 22, 28, 31, 35–37). In individuals at risk for psychosis, all the five negative studies used explicit emotion processing paradigms (20, 39–42). This observation may be explained by the fact that explicit emotion paradigms, such as emotional facial expression recognition paradigms, are more cognitively demanding than implicit emotion paradigms, such as those asking participants to identify the gender of the facial stimuli. Alternatively, the failure to detect increased brain reactivity in these studies (in schizophrenia and individuals “at risk”) may be explained by a lack of power, since 11 of these 13 fMRI studies used event-related rather than block designs (19, 20, 22, 28, 31, 35, 37, 39–42). Of interest, all the studies which reported hippocampal hyper-activations used implicit paradigms (9, 18, 24, 34).

The aberrant brain reactivity to neutral stimuli has been observed in studies using facial stimuli as well as images (e.g., animals, natural scenes, concrete objects, etc.) taken from the *International Affective Pictures System* (IAPS) (46). However, results were more consistent in studies using facial stimuli. Indeed, in schizophrenia, seven out of nine studies that failed to highlight increased brain reactivity used stimuli that were not (exclusively) human faces (19, 22, 28, 31, 35–37), as well as three out of the five negative studies performed in individuals at risk for psychosis (20, 41, 42). This observation suggests that human faces convey information not present in non-facial visual stimuli, which may elicit aberrant brain reactivity in psychotic individuals. At the moment, the available literature does not allow to determine if aberrant brain reactivity is also present in schizophrenia/psychosis in response to non-visual neutral stimuli (e.g., heard words, sounds, etc.).

Lastly, to determine the impact of the patients’ sex on results, we performed a median split of studies based on the percentage of males included in the schizophrenia group of each studies. By doing so, we found no significant influence on sex on neuroimaging outcomes. However, it must be mentioned that only one study explicitly examined the impact of sex differences on the aberrant brain reactivity to neutral stimuli in schizophrenia (25).

## Explanatory Models

To explain the increased brain reactivity to neutral stimuli observed in schizophrenia, most authors have referred to the highly influential aberrant salience hypothesis of psychosis (21). According to this hypothesis, the *positive* symptoms of schizophrenia (e.g., loss of contact with reality) result from the aberrant attribution of motivational value to irrelevant stimuli, due to increased phasic dopamine release in sub-cortical regions (21). According to this model, delusional thinking would emerge from a cognitive attempt to organize these aberrantly salient experiences. Consistently with this model, three studies found associations between increased brain reactivity to neutral stimuli and positive symptoms in schizophrenia (18, 27, 31); however, two studies have found an association between brain reactivity to neutral stimuli and negative symptoms in schizophrenia (16, 26), and only a paucity of studies (*n* = 7) have examined potential associations with psychiatric symptoms. Although the results described in the current review are consistent with the aberrant salience hypothesis, one of the implicit assumptions of this hypothesis is that the chaotic attribution of motivational value to irrelevant stimuli is not explained by the subtle characteristics of stimuli, and that nearly any kind of stimuli can elicit these aberrant experiences.

The case of human faces is interesting in that regard. Although faces used in experimental studies can be *neutral* in their expressed emotion, it is impossible to rule out that even emotionally neutral faces are neutral in every other aspect. Based on recent work performed in social neuroscience, we now know that *emotionally* neutral faces convey subtle information that is implicitly processed by the brain (e.g., physiognomic features such as mouth curvature, distance between the eyes and fullness of lips, as well as traits like masculinity and typicality) (47, 48). Indeed, when watching *emotionally* neutral faces, humans make several spontaneous judgments of which they are barely aware of, including evaluations of trustworthiness, attractiveness, dominance, and intro-/extra-version (47). Thus far, trustworthiness judgments are the ones having received the greatest attention in schizophrenia research. Importantly, preliminary studies have shown that SCZ patients under-estimate the trustworthiness of human faces (49). Based on these findings, a few fMRI studies have examined how schizophrenia patients process (emotionally neutral) faces while judging their trustworthiness. Pinkham et al. (50) showed in two studies that paranoid schizophrenia patients have abnormal amygdala activity when they are processing faces judged as being untrustworthy, compared to controls and non-paranoid schizophrenia patients. Finally, Mukherjee et al. (51) showed that the connectivity between the amygdala and the insular cortex is reduced in schizophrenia patients, compared to controls, when making approachability judgments. Regardless of the heuristic value of the work, it cannot *fully* explain why the brain reactivity of schizophrenia patients to neutral stimuli is increased, simply because these aberrant brain responses have been observed not only in fMRI studies using human faces, but also in several studies using IAPS images (although less consistently) (Table 1), which comprise several types of stimuli other than human faces (e.g., natural scenes, animals, objects, complex social situations, etc.).

Alternatively, some fMRI studies have investigated how neutral stimuli may acquire aberrant significance *via* impaired associative learning. This possibility has been examined in two studies using aversive pavlovian conditioning paradigms (24, 31). In response to conditioned stimuli paired to unconditioned *neutral* stimuli, schizophrenia patients displayed increased activations in emotionally relevant brain regions, such as the ventral striatum and the hippocampus (24). The results of this study tentatively suggest that schizophrenia patients confer aberrant salience to irrelevant stimuli *via* the learning of inappropriate associations. However, the study of Romaniuk et al. did not find increased activations in schizophrenia in response to neutral stimuli (31).

The fact that schizophrenia patients confer emotional salience to irrelevant stimuli may also be explained by a priming effect, reflecting a negative evaluative bias. This hypothesis has been investigated in three fMRI studies using prime faces being presented for a time span too short (e.g., 33 ms) to be consciously processed by participants, followed by target (neutral) faces presented during a longer time span. While Lindner et al. (26) showed that schizophrenia patients have increased amygdala activations when the prime faces express *emotions*, Suslow et al. (33) demonstrated that these hyper-activations are present in schizophrenia when the prime faces have a *neutral* expression, and Rauch et al. (29) did not observe increased activations in schizophrenia. The results of these studies make it difficult to determine if the increased brain responses of schizophrenia patients to neutral stimuli are explained by a priming effect or not.

Finally, the aberrant brain reactivity to irrelevant stimuli in schizophrenia may be explained by reduced neural habituation to emotionally neutral faces (52), but this possibility has been examined in only one study (32).

## Limitations

A few methodological issues limit the interpretation of the available literature. The first issue has to do with the difficulty to retrieve all the functional neuroimaging studies having examined the brain activity of schizophrenia or psychosis-prone individuals during the neutral condition specifically. It is possible that the studies that did not report the results for this experimental condition were precisely the ones that did not find significant between-group differences. Thus, we cannot exclude that the results described here are partially confounded by a reporting bias. However, to minimize this bias, we retrieved all relevant papers instead of relying on abstracts.

Another issue is that all fMRI studies performed in patients diagnosed with schizophrenia have included patients on antipsychotics. Only one study was performed in drug-free patients (28), and none was performed in drug-naive patients. This means that we cannot rule out the possibility that the aberrant brain reactivity of schizophrenia patients to neutral stimuli is brought about by the use of antipsychotics. This is very unlikely however, given that: (i) dopamine plays a key role in motivational salience (53); (ii) striatal dopamine release is increased in schizophrenia and positively correlated with the positive symptoms of the disorder (54); and (ii) antipsychotics are dopamine-D_2_ receptor blockers in the striatum (55). If anything, the fact that schizophrenia patients were treated with antipsychotics may have masked some effects, and this may explain why some studies have failed to detect increased brain reactivity to neutral stimuli in psychotic individuals (19, 20). At the other end of the spectrum, only one study examined how treatment-resistant schizophrenia patients respond to emotionally neutral stimuli (16), although 25–30% of schizophrenia are resistant to antipsychotics, and that treatment resistance is associated with substance misuse, tobacco smoking, suicide, poor quality of life, and increased medication side effects (56). The study found that treatment-resistant schizophrenia patients have hyper-activations in the dorso-medial prefrontal cortex, a region involved in several higher social cognitive processes (57), compared to a control group of schizophrenia patients and to a group of healthy volunteers.

Another issue is that the validity of the neutral minus baseline contrast (used in most of the studies described in Table 1) rests on the assumption that baseline brain activity in schizophrenia patients is comparable to baseline activity in controls. However, substantial evidence from resting-state fMRI studies reliably shows that the *connectivity* within executive, emotional salience, and default mode networks is significantly disrupted in schizophrenia (58, 59). More importantly in the current context, Taylor et al. (60) used positron emission tomography to measure cerebral blood flow in schizophrenia and found that the baseline (tonic) brain activity was increased in the (ventral) striatum and the amygdala in schizophrenia. Likewise, Pinkham et al. (61) and Scheef et al. (62) recently used arterial spin labeling to measure cerebral blood flow in schizophrenia, and both studies found that the amygdala activity was increased at rest in schizophrenia patients, compared to healthy controls [The Scheef et al. (62) study also found hyper-perfusion in the thalamus, the parahippocampus, and the precuneus]. The study of Pinkham et al. (61) is particularly interesting in that it showed that the amygdala was specifically hyper-active at rest in patients with paranoid symptoms. As such, these latter results suggest that a tonic hyper-activity of the amygdala, a limbic region playing a well-documented role in fear processing (63), may fuel paranoid ideation in schizophrenia. From a methodological point of view, these results might explain the heterogeneity of findings of the fMRI studies having examined the neural activity in the entire affective network in schizophrenia patients, using the Emotion minus Rest contrast (5). Likewise, these results might also explain why some studies examining the Neutral minus Rest contrast failed to show that there is increased brain reactivity to neutral stimuli in psychotic individuals (19, 20), and why a minority of studies have even reported decreased brain activations in schizophrenia during the Neutral minus Rest contrast (Table 1).

Finally, another limitation is that the potential impact of sex-differences on the aberrant brain reactivity to neutral stimuli has been scarcely studied, although sex-differences are increasingly investigated in neuro-imaging studies in schizophrenia (64). To date, most studies of gross anatomy show more enlarged ventricles and smaller frontal lobes in men than in women with schizophrenia, reflecting normal sexual dimorphism. In comparison, studies of brain asymmetry and specific cortico-limbic structures suggest a disturbance of normal sexual dimorphism. The relevance of this question is further justified by the findings from Blackford et al. (52), who recently observed that schizophrenia patients have increased limbic (e.g., amygdala and hippocampus) activations when viewing emotionally neutral faces of the opposite sex, but not faces of the same sex.

## Future Perspectives

The available literature suggests that increased brain reactivity to neutral stimuli is fairly common in schizophrenia patients, but that the regions involved vary considerably from one study to another, apart from the amygdala. Prefrontal and cingulate sub-regions as well as the hippocampus may also be involved. By contrasts, results in individuals at risk for psychosis are less consistent. However, smaller effects are to be expected in these “at risk” individuals, and studies performed to date in these individuals may have not been adequately powered to detect such smaller effects. In the future, fMRI studies on the topic will need to be performed in larger samples of individuals at high-risk of developing schizophrenia, in first-episode of psychosis patients, as well as in patients who are drug free and drug naive. The longitudinal effects of antipsychotics on this aberrant brain reactivity will also need to be determined, as well as the potential implication of sex/gender. Methodology-wise, cerebral blood flow at rest will need to be measured in future investigations. Also, it will need to be determined if the aberrant brain reactivity to neutral stimuli is associated with the positive symptoms of schizophrenia or not. Finally, greater attention will need to be paid to non-visual stimuli, as well as to characteristics of faces other than their emotional expressions.

## Author Contributions

SP wrote the manuscript. AT and AM provided several critical comments. Both authors approved the submission of the final version of the manuscript.

## Conflict of Interest Statement

The authors declare that the mini review was conducted in the absence of any commercial or financial relationships that could be construed as a potential conflict of interest.

## References

[1] Foussias G Siddiqui I Fervaha G Agid O Remington G. Dissecting negative symptoms in schizophrenia: opportunities for translation into new treatments. J Psychopharmacol (2015) 29(2):116–26. 10.1177/0269881114562092 25516370

[2] Cohen AS Minor KS. Emotional experience in patients with schizophrenia revisited: meta-analysis of laboratory studies. Schizophr Bull (2010) 36(1):143–50. 10.1093/schbul/sbn061 18562345 PMC2800132

[3] Llerena K Strauss GP Cohen AS. Looking at the other side of the coin: a meta-analysis of self-reported emotional arousal in people with schizophrenia. Schizophr Res (2012) 142(1):65–70. 10.1016/j.schres.2012.09.005 23040736 PMC3502689

[4] Li H Chan RC McAlonan GM Gong QY. Facial emotion processing in schizophrenia: a meta-analysis of functional neuroimaging data. Schizophr Bull (2010) 36(5):1029–39. 10.1093/schbul/sbn190 19336391 PMC2930350

[5] Anticevic A Van Snellenberg JX Cohen RE Repovs G Dowd EC Barch DM. Amygdala recruitment in schizophrenia in response to aversive emotional material: a meta-analysis of neuroimaging studies. Schizophr Bull (2012) 38(3):608–21. 10.1093/schbul/sbq131 21123853 PMC3329999

[6] Williams LM Das P Harris AW Liddell BB Brammer MJ Olivieri G Dysregulation of arousal and amygdala-prefrontal systems in paranoid schizophrenia. Am J Psychiatry (2004) 161(3):480–9. 10.1176/appi.ajp.161.3.480 14992974

[7] Fernandez-Egea E Parellada E Lomena F Falcon C Pavia J Mane A 18FDG PET study of amygdalar activity during facial emotion recognition in schizophrenia. Eur Arch Psychiatry Clin Neurosci (2010) 260(1):69–76. 10.1007/s00406-009-0020-6 19452191

[8] Hall J Whalley HC McKirdy JW Romaniuk L McGonigle D McIntosh AM Overactivation of fear systems to neutral faces in schizophrenia. Biol Psychiatry (2008) 64(1):70–3. 10.1016/j.biopsych.2007.12.014 18295746

[9] Holt DJ Kunkel L Weiss AP Goff DC Wright CI Shin LM Increased medial temporal lobe activation during the passive viewing of emotional and neutral facial expressions in schizophrenia. Schizophr Res (2006) 82(2–3):153–62. 10.1016/j.schres.2005.09.021 16377154

[10] Mier D Lis S Zygrodnik K Sauer C Ulferts J Gallhofer B Evidence for altered amygdala activation in schizophrenia in an adaptive emotion recognition task. Psychiatry Res (2014) 221(3):195–203. 10.1016/j.pscychresns.2013.12.001 24434194

[11] Mier D Sauer C Lis S Esslinger C Wilhelm J Gallhofer B Neuronal correlates of affective theory of mind in schizophrenia out-patients: evidence for a baseline deficit. Psychol Med (2010) 40(10):1607–17. 10.1017/S0033291709992133 20056024

[12] Shin NY Park HY Jung WH Park JW Yun JY Jang JH Effects of oxytocin on neural response to facial expressions in patients with schizophrenia. Neuropsychopharmacology (2015) 40(9):2286 10.1038/npp.2015.78 26174494 PMC4613623

[13] Bjorkquist OA Herbener ES. Social perception in schizophrenia: evidence of temporo-occipital and prefrontal dysfunction. Psychiatry Res (2013) 212(3):175–82. 10.1016/j.pscychresns.2012.12.002 23642469

[14] Habel U Chechko N Pauly K Koch K Backes V Seiferth N Neural correlates of emotion recognition in schizophrenia. Schizophr Res (2010) 122(1–3):113–23. 10.1016/j.schres.2010.06.009 20663646

[15] Mothersill O Morris DW Kelly S Rose EJ Bokde A Reilly R Altered medial prefrontal activity during dynamic face processing in schizophrenia spectrum patients. Schizophr Res (2014) 157(1–3):225–30. 10.1016/j.schres.2014.05.023 24888525

[16] Potvin S Tikasz A Lungu O Dumais A Stip E Mendrek A. Emotion processing in treatment-resistant schizophrenia patients treated with clozapine: an fMRI study. Schizophr Res (2015) 168(1–2):377–80. 10.1016/j.schres.2015.07.046 26255082

[17] Seiferth NY Pauly K Habel U Kellermann T Shah NJ Ruhrmann S Increased neural response related to neutral faces in individuals at risk for psychosis. Neuroimage (2008) 40(1):289–97. 10.1016/j.neuroimage.2007.11.020 18187342

[18] Surguladze S Russell T Kucharska-Pietura K Travis MJ Giampietro V David AS A reversal of the normal pattern of parahippocampal response to neutral and fearful faces is associated with reality distortion in schizophrenia. Biol Psychiatry (2006) 60(5):423–31. 10.1016/j.biopsych.2005.11.021 16487943

[19] Lee SK Chun JW Lee JS Park HJ Jung YC Seok JH Abnormal neural processing during emotional salience attribution of affective asymmetry in patients with schizophrenia. PLoS One (2014) 9(3):e90792. 10.1371/journal.pone.0090792 24619004 PMC3949688

[20] van Buuren M Vink M Rapcencu AE Kahn RS. Exaggerated brain activation during emotion processing in unaffected siblings of patients with schizophrenia. Biol Psychiatry (2011) 70(1):81–7. 10.1016/j.biopsych.2011.03.011 21531384

[21] Kapur S. Psychosis as a state of aberrant salience: a framework linking biology, phenomenology, and pharmacology in schizophrenia. Am J Psychiatry (2003) 160(1):13–23. 10.1176/appi.ajp.160.1.13 12505794

[22] Dowd EC Barch DM. Anhedonia and emotional experience in schizophrenia: neural and behavioral indicators. Biol Psychiatry (2010) 67(10):902–11. 10.1016/j.biopsych.2009.10.020 20004364 PMC3113677

[23] Holt DJ Lakshmanan B Freudenreich O Goff DC Rauch SL Kuperberg GR. Dysfunction of a cortical midline network during emotional appraisals in schizophrenia. Schizophr Bull (2011) 37(1):164–76. 10.1093/schbul/sbp067 19605517 PMC3004194

[24] Jensen J Willeit M Zipursky RB Savina I Smith AJ Menon M The formation of abnormal associations in schizophrenia: neural and behavioral evidence. Neuropsychopharmacology (2008) 33(3):473–9. 10.1038/sj.npp.1301437 17473838

[25] Lakis N Mendrek A. Individuals diagnosed with schizophrenia assign emotional importance to neutral stimuli: an FMRI study. ISRN Psychiatry (2013) 2013:965428. 10.1155/2013/965428 24381781 PMC3871502

[26] Lindner C Dannlowski U Bauer J Ohrmann P Lencer R Zwitserlood P Affective flattening in patients with schizophrenia: differential association with amygdala response to threat-related facial expression under automatic and controlled processing conditions. Psychiatry Investig (2016) 13(1):102–11. 10.4306/pi.2016.13.1.102 26766952 PMC4701673

[27] Modinos G Tseng HH Falkenberg I Samson C McGuire P Allen P. Neural correlates of aberrant emotional salience predict psychotic symptoms and global functioning in high-risk and first-episode psychosis. Soc Cogn Affect Neurosci (2015) 10(10):1429–36. 10.1093/scan/nsv035 25809400 PMC4590543

[28] Pankow A Friedel E Sterzer P Seiferth N Walter H Heinz A Altered amygdala activation in schizophrenia patients during emotion processing. Schizophr Res (2013) 150(1):101–6. 10.1016/j.schres.2013.07.015 23911256

[29] Rauch AV Reker M Ohrmann P Pedersen A Bauer J Dannlowski U Increased amygdala activation during automatic processing of facial emotion in schizophrenia. Psychiatry Res (2010) 182(3):200–6. 10.1016/j.pscychresns.2010.03.005 20488680

[30] Reske M Habel U Kellermann T Backes V Jon Shah N von Wilmsdorff M Differential brain activation during facial emotion discrimination in first-episode schizophrenia. J Psychiatr Res (2009) 43(6):592–9. 10.1016/j.jpsychires.2008.10.012 19056093

[31] Romaniuk L Honey GD King JR Whalley HC McIntosh AM Levita L Midbrain activation during Pavlovian conditioning and delusional symptoms in schizophrenia. Arch Gen Psychiatry (2010) 67(12):1246–54. 10.1001/archgenpsychiatry.2010.169 21135324

[32] Schwartz BL Vaidya CJ Shook D Deutsch SI. Neural basis of implicit memory for socio-emotional information in schizophrenia. Psychiatry Res (2013) 206(2–3):173–80. 10.1016/j.psychres.2012.10.005 23123045 PMC3586761

[33] Suslow T Lindner C Dannlowski U Walhofer K Rodiger M Maisch B Automatic amygdala response to facial expression in schizophrenia: initial hyperresponsivity followed by hyporesponsivity. BMC Neurosci (2013) 14:140. 10.1186/1471-2202-14-140 24219776 PMC3832234

[34] Taylor SF Chen AC Tso IF Liberzon I Welsh RC. Social appraisal in chronic psychosis: role of medial frontal and occipital networks. J Psychiatr Res (2011) 45(4):526–38. 10.1016/j.jpsychires.2010.08.004 20797730 PMC2994990

[35] Taylor SF Welsh RC Chen AC Velander AJ Liberzon I. Medial frontal hyperactivity in reality distortion. Biol Psychiatry (2007) 61(10):1171–8. 10.1016/j.biopsych.2006.11.029 17434455

[36] Taylor SF Liberzon I Decker LR Koeppe RA. A functional anatomic study of emotion in schizophrenia. Schizophr Res (2002) 58(2–3):159–72. 10.1016/S0920-9964(01)00403-0 12409155

[37] Ursu S Kring AM Gard MG Minzenberg MJ Yoon JH Ragland JD Prefrontal cortical deficits and impaired cognition-emotion interactions in schizophrenia. Am J Psychiatry (2011) 168(3):276–85. 10.1176/appi.ajp.2010.09081215 21205806 PMC4019338

[38] Whalley HC McKirdy J Romaniuk L Sussmann J Johnstone EC Wan HI Functional imaging of emotional memory in bipolar disorder and schizophrenia. Bipolar Disord (2009) 11(8):840–56. 10.1111/j.1399-5618.2009.00768.x 19922553

[39] Barbour T Murphy E Pruitt P Eickhoff SB Keshavan MS Rajan U Reduced intra-amygdala activity to positively valenced faces in adolescent schizophrenia offspring. Schizophr Res (2010) 123(2–3):126–36. 10.1016/j.schres.2010.07.023 20716480 PMC3174012

[40] Diwadkar VA Wadehra S Pruitt P Keshavan MS Rajan U Zajac-Benitez C Disordered corticolimbic interactions during affective processing in children and adolescents at risk for schizophrenia revealed by functional magnetic resonance imaging and dynamic causal modeling. Arch Gen Psychiatry (2012) 69(3):231–42. 10.1001/archgenpsychiatry.2011.1349 22393216 PMC7988184

[41] Modinos G Ormel J Aleman A. Altered activation and functional connectivity of neural systems supporting cognitive control of emotion in psychosis proneness. Schizophr Res (2010) 118(1–3):88–97. 10.1016/j.schres.2010.01.030 20188516

[42] van der Velde J Opmeer EM Liemburg EJ Bruggeman R Nieboer R Wunderink L Lower prefrontal activation during emotion regulation in subjects at ultrahigh risk for psychosis: an fMRI-study. npj Schizophrenia (2015) 1:15026 10.1038/npjschz.2015.26 PMC4849453 27336036

[43] Fusar-Poli P Placentino A Carletti F Landi P Allen P Surguladze S Functional atlas of emotional faces processing: a voxel-based meta-analysis of 105 functional magnetic resonance imaging studies. J Psychiatry Neurosci (2009) 34(6):418–32. 19949718 PMC2783433

[44] Nees F Pohlack ST. Functional MRI studies of the hippocampus. Front Neurol Neurosci (2014) 34:85–94. 10.1159/000356427 24777133

[45] Winton-Brown TT Fusar-Poli P Ungless MA Howes OD. Dopaminergic basis of salience dysregulation in psychosis. Trends Neurosci (2014) 37(2):85–94. 10.1016/j.tins.2013.11.003 24388426

[46] Lang P Bradley M Cuthbert B International Affective Picture System (IAPS): Affective Ratings of Pictures and Instruction Manual. Gainesville, FL: University of Florida (2008). Technical Report A-8.

[47] Todorov A Baron SG Oosterhof NN. Evaluating face trustworthiness: a model based approach. Soc Cogn Affect Neurosci (2008) 3(2):119–27. 10.1093/scan/nsn009 19015102 PMC2555464

[48] Todorov A Olivola CY Dotsch R Mende-Siedlecki P. Social attributions from faces: determinants, consequences, accuracy, and functional significance. Annu Rev Psychol (2015) 66:519–45. 10.1146/annurev-psych-113011-143831 25196277

[49] Tremeau F Antonius D Todorov A Rebani Y Ferrari K Lee SH Implicit emotion perception in schizophrenia. J Psychiatr Res (2015) 71:112–9. 10.1016/j.jpsychires.2015.10.001 26473695

[50] Pinkham AE Hopfinger JB Ruparel K Penn DL. An investigation of the relationship between activation of a social cognitive neural network and social functioning. Schizophr Bull (2008) 34(4):688–97. 10.1093/schbul/sbn031 18477583 PMC2632458

[51] Mukherjee P Whalley HC McKirdy JW Sprengelmeyer R Young AW McIntosh AM Altered amygdala connectivity within the social brain in schizophrenia. Schizophr Bull (2014) 40(1):152–60. 10.1093/schbul/sbt086 23851067 PMC3885300

[52] Blackford JU Williams LE Heckers S. Neural correlates of out-group bias predict social impairment in patients with schizophrenia. Schizophr Res (2015) 164(1–3):203–9. 10.1016/j.schres.2015.03.019 25864952 PMC4512167

[53] Berridge KC Robinson TE. What is the role of dopamine in reward: hedonic impact, reward learning, or incentive salience? Brain Res Brain Res Rev (1998) 28(3):309–69. 10.1016/S0165-0173(98)00019-8 9858756

[54] Howes OD Kambeitz J Kim E Stahl D Slifstein M Abi-Dargham A The nature of dopamine dysfunction in schizophrenia and what this means for treatment. Arch Gen Psychiatry (2012) 69(8):776–86. 10.1001/archgenpsychiatry.2012.169 22474070 PMC3730746

[55] Seeman P Atypical antipsychotics: mechanism of action. Can J Psychiatry (2002) 47(1):27–38. 11873706

[56] Kennedy JL Altar CA Taylor DL Degtiar I Hornberger JC. The social and economic burden of treatment-resistant schizophrenia: a systematic literature review. Int Clin Psychopharmacol (2014) 29(2):63–76. 10.1097/YIC.0b013e32836508e6 23995856

[57] Eickhoff SB Laird AR Fox PT Bzdok D Hensel L. Functional segregation of the human dorsomedial prefrontal cortex. Cereb Cortex (2016) 26(1):304–21. 10.1093/cercor/bhu250 25331597 PMC4677979

[58] Cao H Dixson L Meyer-Lindenberg A Tost H. Functional connectivity measures as schizophrenia intermediate phenotypes: advances, limitations, and future directions. Curr Opin Neurobiol (2016) 36:7–14. 10.1016/j.conb.2015.07.008 26276700

[59] Sheffield JM Barch DM. Cognition and resting-state functional connectivity in schizophrenia. Neurosci Biobehav Rev (2016) 61:108–20. 10.1016/j.neubiorev.2015.12.007 26698018 PMC4731300

[60] Taylor SF Phan KL Britton JC Liberzon I. Neural response to emotional salience in schizophrenia. Neuropsychopharmacology (2005) 30(5):984–95. 10.1038/sj.npp.1300679 15689961

[61] Pinkham AE Liu P Lu H Kriegsman M Simpson C Tamminga C. Amygdala hyperactivity at rest in paranoid individuals with schizophrenia. Am J Psychiatry (2015) 172(8):784–92. 10.1176/appi.ajp.2014.14081000 25815418

[62] Scheef L Manka C Daamen M Kuhn KU Maier W Schild HH Resting-state perfusion in nonmedicated schizophrenic patients: a continuous arterial spin-labeling 3.0-T MR study. Radiology (2010) 256(1):253–60. 10.1148/radiol.10091224 20505069

[63] Phelps EA LeDoux JE. Contributions of the amygdala to emotion processing: from animal models to human behavior. Neuron (2005) 48(2):175–87. 10.1016/j.neuron.2005.09.025 16242399

[64] Mendrek A Mancini-Marie A. Sex/gender differences in the brain and cognition in schizophrenia. Neurosci Biobehav Rev (2015). 10.1016/j.neubiorev.2015.10.013 26743859

